# Supplementary material for: Elevated Plasma Apurinic/Apyrimidinic Endonuclease 1/Redox Effector Factor-1 Levels in Refractory Kawasaki Disease
Source: Biomedicines. 2022 Jan 17;10(1):190. doi: 10.3390/biomedicines10010190 (PMC8773471; doi:10.3390/biomedicines10010190)
Supplement: Supplementary file 1 [file biomedicines-10-00190-s001.zip › biomedicines-1464288-supplementary.pdf]

Supplementary Figure S1

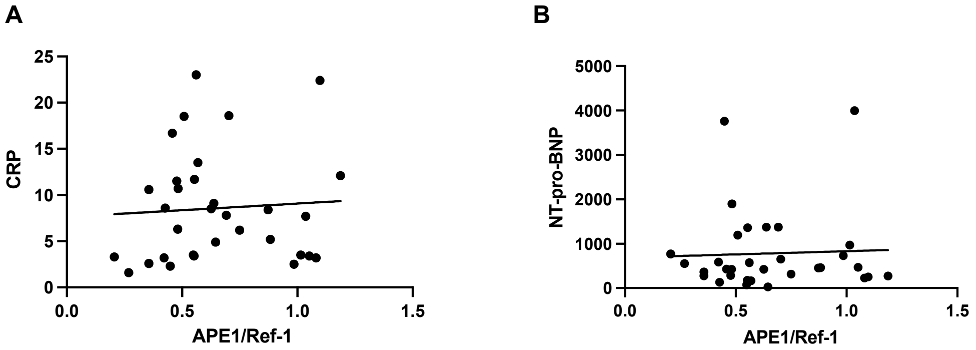

Figure S1. Correlation between plasma APE1/Ref-1 levels and laboratory data. (A) Correlation between APE1/Ref-1 and CRP levels (B) correlation between APE1/Ref-1 and NT-proBNP levels.

Statistical significance was determined via Pearson's correlation analysis.
